# Occupational Fatalities During the Oil and Gas Boom — United States, 2003–2013

**Published:** 2015-05-29

**Authors:** Krystal L. Mason, Kyla D. Retzer, Ryan Hill, Jennifer M. Lincoln

**Affiliations:** 1 Office of Administrative and Management Services, National Institute for Occupational Safety and Health, CDC

During 2003–2013, the U.S. oil and gas extraction industry experienced unprecedented growth, doubling the size of its workforce and increasing the number of drilling rigs by 71% (1,2). To describe fatal events among oil and gas workers during this period, CDC analyzed data from the Bureau of Labor Statistics (BLS) Census of Fatal Occupational Injuries (CFOI), a comprehensive database of fatal work injuries (3). During 2003–2013, the number of work-related fatalities in the oil and gas extraction industry increased 27.6%, with a total of 1,189 deaths; however, the annual occupational fatality rate significantly decreased 36.3% (p<0.05) during this 11-year period. Two-thirds of all worker fatalities were attributed to transportation incidents (479, [40.3%]) and contact with objects/equipment (308 [25.9%]). More than 50% of persons fatally injured were employed by companies that service wells (615 [51.7%]). It is important for employers to consider measures such as land transportation safety policies and engineering controls (e.g., automated technologies) that would address these leading causes of death and reduce workers’ exposure to hazards (4–6).

Publicly available data from CFOI were used to determine the number of fatal injuries to workers in the U.S. land-based and offshore oil and gas extraction industry during 2003–2013. CFOI collects information from multiple data sources to identify, verify, and describe fatal work injuries (3). According to CFOI, a fatal injury is considered work-related if 1) the event leading to the injury occurred while the employee was working and 2) the event is verified by at least two independent data sources.* The North American Industry Classification System (NAICS) was used to identify fatal events among the three types of companies in the oil and gas extraction industry: oil and gas operators that control and manage leased areas (NAICS 211), drilling contractors that drill the wells (NAICS 213111), and well-servicing companies that provide all other types of support operations that prepare a well for production and completion (NAICS 213112).

Annual occupational fatal injury rates were calculated using worker estimates from the BLS Quarterly Census of Employment and Wages (1). Annual and overall fatality rates were also calculated by event type according to the Occupational Injury and Illness Classification System and by company type using NAICS. Negative binomial regression was used to estimate rates. The percent rate of change, incident rate ratio, and corresponding confidence intervals were calculated for the 11-year period, the five most frequent fatal events, and by company type. Each company and event type represents separate regression models.

During 2003–2013, 1,189 oil and gas extraction industry employees died while working, resulting in an average of 108 deaths per year and an annual average occupational fatality rate of 25.0 deaths per 100,000 workers. The highest fatality rate occurred in 2006 (32.4 deaths per 100,000 workers) with 125 fatalities (Figure). During this period all but 10 decedents were male, and the largest numbers of deaths were to workers aged 25–34 years (331 [27.8%]). Most were non-Hispanic whites (844 [71.0%]). Two-thirds of the fatalities were attributed to transportation incidents (479 [40.3%]) and contact with objects/equipment (308 [25.9%]). Incidents on land (as opposed to air or water) made up 86.2% of the transportation events. The remainder of the most frequent events were the result of fires or explosions (170, [14.3%]); exposure to harmful substances or environments (105 [8.8%]); or falls, slips, and trips (97 [8.2%]). The largest number of fatalities occurred among workers employed by well-servicing companies (615), followed by drilling contractors (378), and operators (196); but the highest fatality rate was among workers employed by drilling companies (44.6 per 100,000 workers), followed by well-servicing companies (27.9), and operators (11.6) (Table 1).

Although the oil and gas extraction industry’s number of occupational fatalities increased 27.6% during the 11-year period, it did not increase as much as the number of workers, resulting in a significant decrease in the fatality rate of 36.3% (Table 2). The average annual decrease was 4% per year (Table 1). Oil and gas operators experienced the largest decrease in the rate of fatal injuries, 8% per year (p<0.01), followed by well-servicing companies (4% per year, p<0.05). Among event types, contact with objects/equipment experienced the greatest decrease, 9% per year (p<0.001); transportation events also showed a significant decrease, 3% per year (p<0.05).

## Discussion

Previous research found a positive correlation between the level of activity (number of active drilling rigs) and the occupational fatality rate in the U.S. oil and gas extraction industry (7). This report found that although the number of active drilling rigs increased by 71% and the number of oil and gas extraction workers more than doubled (1,2) during 2003–2013, the industry’s fatality rate significantly decreased.

Transportation events and contact with objects/equipment events were the most frequent fatal events in the oil and gas extraction industry, which is consistent with previously reported data (7,8). This analysis showed the rate of fatalities caused by contact with objects/equipment experienced the greatest decrease during 2003–2013 (p<0.001), which might be related to the increased use of automated technologies on drilling rigs such as hydraulic catwalks to move drill pipe from ground level to the rig floor and powered tongs used to make and break drilling pipe connections. A recent study found lower non-fatal injury rates on rigs with automated technologies designed to reduce workers’ exposure to hazardous equipment (9). This report also found that the transportation-related fatality rate decreased significantly (p<0.05) despite an increase in the number of fatalities. Previous research showed the majority of transportation fatalities were the result of motor vehicle crashes killing occupants of light trucks (e.g., pickup trucks), which are largely unregulated (8). Transportation fatalities did not include deaths while commuting to and from work, as these are not typically considered work-related. However, frequent long distance commutes are common for workers in this industry and are an area of concern.

Collaboration between industry, government, and academic institutions might have contributed to improved safety for workers and likely should continue to drive the fatality rate further down. In 2003, the National Service, Transmission, Exploration and Production Safety Network was founded in South Texas by the Occupational Safety and Health Administration and industry to share best practices in oil and gas safety and health. Since then, the organization has expanded to 22 independent networks serving 15 oil and gas producing states. Another group, the National Occupational Research Agenda Oil and Gas Extraction Sector Council, was created by CDC in 2008 as a partnership program to establish an occupational safety and health research agenda. Since then, the council has created several safety products targeting high-risk workers and activities (10). In addition, regional groups, such as the Appalachian Shale Transportation Safety Workgroup, have formed to identify and share best practices in transportation safety.

This report is subject to at least three limitations. First, it would have been preferable to calculate fatality rates using estimates of the number of full-time equivalent workers, which takes overtime into consideration, but these estimates were not available for this industry. Second, changes made to the Occupational Injury and Illness Classification System for years 2011 and later are considered a break in series. Although event-type categories reported here did not undergo significant change in 2011, clarifications in the order of precedence for the event type categories were issued that might have led to differences in event coding starting in 2011. Lastly, fatal event numbers for 2013 are preliminary and might be incomplete. Historically, transportation event data are the most incomplete, and this could affect the trend.

What is already known on this topic?

Fatality rates for workers in the oil and gas extraction industry have historically been higher than the rate for all workers (an average of seven times higher every year since 2003, when fatality rates for oil and gas workers were first added to the data collected). During 2003–2013, an oil and gas boom occurred and the industry doubled its workforce and experienced a 71% increase in active drilling rigs. Although the number of fatal injuries also increased during this time, trends in fatality rates during this boom have not been previously reported.

What is added by this report?

The fatality rate for the oil and gas extraction industry decreased by 36.3% (p<0.001) during 2003–2013, from 29.0 to 19.1 per 100,000 workers per year. The rate for fatalities caused by contact with objects and equipment experienced the greatest decrease (60.8%, p<0.001). Transportation incidents continue to be the leading cause of death.

What are the implications for public health practice?

It is important for oil and gas industry employers to continue to implement safety measures that target causes of the most frequent fatal events, including a land transportation safety policy for all workers who drive as a part of their duties. To target injury prevention programs, it is important that occupational safety and health researchers continue and enhance surveillance efforts to identify and report on risk factors for different types of fatal injuries among different sectors of the oil and gas extraction industry.

Although the fatality rate in the oil and gas extraction industry remains an average of seven times higher than among U.S. workers in general (25.1 compared with 3.7 per 100,000 per year), the oil and gas extraction industry has achieved a substantial decrease in fatality rates in recent years. It is important for oil and gas industry employers to continue implementation of safety measures that target causes of the most frequent fatal events. One example is having a land transportation safety policy that outlines safety procedures for all workers who drive as a part of their duties. Another example is adoption of automated technologies that reduce workers’ exposure to oil rig hazards. Occupational safety and health researchers need to continue and enhance surveillance efforts and identify risk factors for different types of fatal injuries among different sectors of the oil and gas extraction industry. The data from surveillance efforts will be useful to industry safety and health networks and can be used to create targeted interventions to reduce worker fatalities.

## Figures and Tables

**FIGURE:**
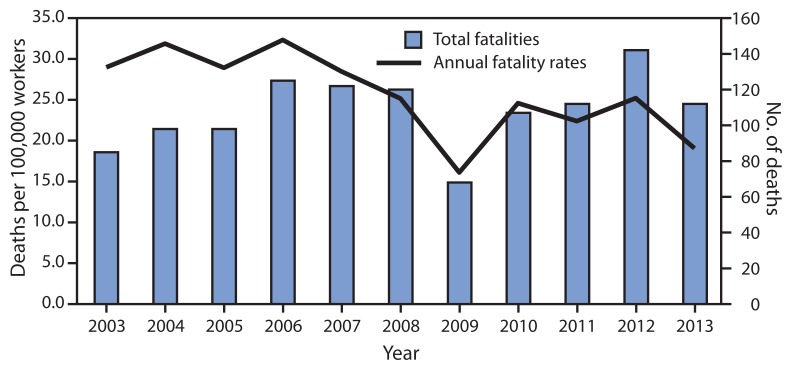
Number* and rate^†^ of fatal injuries among workers in the oil and gas extraction industry, by year — United States, 2003–2013^§^ **Sources:** U.S. Department of Labor, Bureau of Labor Statistics, Census of Fatal Occupational Injuries (2003–2013) and U.S. Department of Labor, Bureau of Labor Statistics, Quarterly Census of Employment and Wages (2003–2013). * N = 1,189. ^†^ Fatality rate calculated per 100,000 workers; significant decrease in fatality rate during 2003–2013 (negative binomial regression chi-square = 0.057; p<0.01). ^§^ 2013 data are preliminary.

**TABLE 1:** Trends* in worker fatality rates in the oil and gas extraction industry, by company type and event type, using an unadjusted model — United States, 2003–2013†

Company/event type	No.	(%)	Fatality rate§	% Rate change¶	IRR	(95% CI)	p-value
**Total Fatalities**	**1,189**		**25.0**	**−36.3**	**0.956**	**(0.932–0.980)**	**0.000** **
**By company (NAICS code)**							
Operators (211)	196	(16.5)	11.6	−58.2	0.917	(0.869–0.967)	0.001**
Drilling contractors (213111)	378	(31.8)	44.6	−27.2	0.969	(0.931–1.008)	0.118
Well-servicing companies (213112)	615	(51.7)	27.9	−33.4	0.960	(0.962–0.996)	0.028**
**By event** ††							
Transportation	479	(40.3)	10.1	−28.1	0.968	(0.938–0.998)	0.040**
Contact with objects/equipment	308	(25.9)	6.5	−60.8	0.910	(0.879–0.944)	0.000**
Fires/explosions§§	170	(14.3)	3.6	−41.3	0.948	(0.884–1.017)	0.137
Exposure to harmful environments/substances§§	104	(8.7)	2.2	−42.6	0.946	(0.890–1.006)	0.076
Falls§§	97	(8.2)	2.0	+26.8	1.024	(0.960–1.093)	0.469

**Sources:** U.S. Department of Labor, Bureau of Labor Statistics, Census of Fatal Occupational Injuries (2003–2013) and U.S. Department of Labor, Bureau of Labor Statistics, Quarterly Census of Employment and Wages (2003–2013).

**Abbreviations:** CI = confidence interval; IRR = incident rate ratio; NAICS = North American Industry Classification System.

* Determined by negative binomial regression analyses.

† Data for 2013 are preliminary.

§ Annual average fatality rate per 100,000 workers.

¶ Using predicted values from negative binomial regressions over 11 years.

** Statistically significant at p<0.05.

†† Break in Occupational Injury and Illness Classification System series in 2011.

§§ Contain one or more years during which the number of fatalities was <10.

**TABLE 2:** Annual fatality rates among workers in the oil and gas extraction industry, by company type and event type — United States, 2003–2013*†

	Fatality rates (yr)											
		
Company/event type	2003	2004	2005	2006	2007	2008	2009	2010	2011	2012	2013	% Rate change§
**Total**	**29.0**	**31.9**	**29.0**	**32.4**	**28.5**	**25.2**	**16.1**	**24.6**	**22.4**	**24.5**	**19.1**	**−36.3**
**By company**												
Operators	14.1	23.9	13.5	16.3	10.3	13.1	7.5	7.6	7.6	13.8	6.1	−58.2
Drilling contractors	50.5	52.3	51.0	45.1	49.7	32.4	42.8	63.1	47.0	42.2	25.7	−27.2
Well-servicing companies	34.7	30.4	32.3	39.2	33.0	30.9	14.0	23.8	24.0	27.3	25.6	−33.4
**By event**												
Transportation	10.6	14.7	10.1	11.2	11.7	10.3	6.4	9.4	10.2	11.0	7.5	−28.1
Contact with objects/equipment	8.9	9.5	8.0	10.1	9.4	6.3	5.0	4.8	5.2	4.3	4.3	−60.8
Fires/explosions	6.5	3.6	3.6	5.4	2.3	3.8	1.4¶	5.8	2.4	4.1	2.2	−41.3
Exposure to harmful environments/substances	1.7¶	1.6¶	3.3	3.1	3.5	2.5	2.1¶	2.3	1.8¶	1.4¶	1.4¶	−42.6
Falls	1.4¶	2.6¶	3.0	2.1¶	1.4¶	1.9¶	0.7¶	1.6¶	2.0	3.2	2.4	+26.8

**Sources:** U.S. Department of Labor, Bureau of Labor Statistics, Census of Fatal Occupational Injuries (2003–2013). U.S. Department of Labor, Bureau of Labor Statistics, Quarterly Census of Employment and Wages (2003–2013).

* 2013 data are preliminary.

† Break in Occupational Injury and Illness Classification System series in 2011.

§ Using predicted values from negative binomial regressions over 11 years.

¶ Contain one or more years during which the number of fatalities was <10.

## Footnotes

* Additional information is available at http://www.bls.gov/iif/oshcfdef.htm.
